# Electron Microscopy Views of Dimorphic Chloroplasts in C4 Plants

**DOI:** 10.3389/fpls.2020.01020

**Published:** 2020-07-03

**Authors:** Keith Ka Ki Mai, Peng Gao, Byung-Ho Kang

**Affiliations:** Centre for Cell and Developmental Biology, State Key Laboratory for Agrobiotechnology, School of Life Sciences, The Chinese University of Hong Kong, Hong Kong, Hong Kong

**Keywords:** chloroplast, thylakoid, C4 photosynthesis, single-cell C4 plants, electron microscopy, electron tomography

## Abstract

C4 plants enhance photosynthesis efficiency by concentrating CO_2_ to the site of Rubisco action. Chloroplasts in C4 plants exhibit structural dimorphism because thylakoid architectures vary depending on energy requirements. Advances in electron microscopy imaging capacity and sample preparation technologies allowed characterization of thylakoid structures and their macromolecular arrangements with unprecedented precision mostly in C3 plants. The thylakoid is assembled during chloroplast biogenesis through collaboration between the plastid and nuclear genomes. Recently, the membrane dynamics involved in the assembly process has been investigated with 3D electron microscopy, and molecular factors required for thylakoid construction have been characterized. The two classes of chloroplasts in C4 plants arise from common precursors, but little is known about how a single type of chloroplasts grow, divide, and differentiate to mature into distinct chloroplasts. Here, we outline the thylakoid structure and its assembly processes in C3 plants to discuss ultrastructural analyses of dimorphic chloroplast biogenesis in C4 plant species. Future directions for electron microscopy research of C4 photosynthetic systems are also proposed.

## Introduction

Since the early days of microscopy, chloroplasts have attracted attention from microscopists. They are abundant in leaves, larger than other organelles, and do not require staining for microscopic imaging. As reviewed by Staehelin in 2003, the study of chloroplast structure has advanced with enhancements in light and electron microscopy from two-dimensional transmission electron microscopy (TEM) imaging to three-dimensional (3D) electron tomography (ET) and with improvements in sample preservation from chemically preservation to freeze-fracture to high-pressure freezing (Staehelin, 2003). The innovations in electron microscopy technology have enabled plant cell biologists to monitor structural and functional dynamics of chloroplasts at nanometer resolution in molecular detail (Kirchhoff, 2018).

The earliest 3D model for the complex thylakoid structure in the chloroplast observed in electron micrographs was the helical model proposed by Paolillo and later modified by Brangeon and Mustárdy (Brangeon and Mustardy, 1979; Mustárdy et al., 2008; Lindquist et al., 2016). In this model, the granum stack is joined to the stroma thylakoid by a series of right-handed helices. This model helps explain why the same stroma thylakoid appears to attach to different grana thylakoids in different parts of the grana stack and why it appears shifted in opposite directions at opposite sides of the granum. Shimoni et al. (2005) proposed a different model based on ET data known as the bifurcation model. In this model, the grana stacks consist of repeating units of bifurcations of the stroma lamellae. Later studies employing cryo-electron tomography (cryo-ET), provided evidence in support of the helical model (Austin and Staehelin, 2011; Daum and Kuhlbrandt, 2011). The helical model is now the most widely accepted model for thylakoid architecture in higher plants. It was recently demonstrated that the thylakoid’s helical arrangement is for constituting an interconnected network of densely packed membrane layers using ET analysis and energy minimization modeling of thylakoid membranes (Bussi et al., 2019), as was observed in the helical arrangement of the rough endoplasmic reticulum in secretory cells (Terasaki et al., 2013).

The high-resolution 3D morphological analyses of chloroplasts have been focused on those in *Arabidopsis thaliana* (*Arabidopsis*) and legume species. These plants are classified as C3, and their chloroplasts are thought to be uniform with respect to photosynthesis. By contrast, C4 plants have two types of chloroplasts that suppress photorespiration by the ribulose-1,5-bisphosphate carboxylase/oxygenase (Rubisco) (Edwards et al., 2001). One type of chloroplast fixes CO_2_ to generate four carbon (C4) acids. In the other type of chloroplast, the Calvin cycle occurs, which regenerates CO_2_ from the C4 acids. Cycles of C4 acids pump CO_2_ near Rubisco. These functionally distinct chloroplasts are readily distinguished in electron micrographs (Voznesenskaya et al., 1999).

Because C4 plants grow better than C3 plants under adverse conditions, molecular mechanisms of C4 photosynthesis have been investigated with a goal to introduce merits of C4 plants into C3 plants (Leegood, 2002; Gowik and Westhoff, 2011). C4 grasses include maize, sorghum, and sugarcane, which are valuable, widely cultivated crop plants. Another interesting aspect of C4 photosynthesis is its anatomical variations. C4 cycles were originally thought to operate between two adjacent cells but this notion was challenged when single-cell C4 (SCC4) plants, in which CO_2_ transport occurs within single photosynthetic cells, were discovered (Voznesenskaya et al., 2001; Edwards et al., 2004).

In this review, we summarize the macromolecular organizations of thylakoids in C3 plants and discuss their assembly process during chloroplast biogenesis with an emphasis on what has been learned from electron microscopy/tomography analyses. We will review the link between structures and functions of dimorphic chloroplasts in mature leaves of maize (a C4 plant with Kranz anatomy) and *Bienertia sinuspersici* (a SCC4 plant). These systems were chosen for discussion because diversification of maize and *B. sinuspersici* chloroplasts for the C4 leaf development have has been characterized at the level of electron microscopy/tomography. Further ultrastructural studies to fill gaps in our understanding of the C4 photosynthetic machinery are proposed.

## Macromolecular Constituents of the Thylakoid Membrane

Large protein complexes of the electron transport chain embedded in the thylakoid membrane are differentially distributed in the helical architecture. PSII and its LHCB subunits are confined to the stacked grana region of the helix. They are closely packed in the region, forming semicrystalline arrays. By contrast, ATP synthase and PSI complexes are known to be excluded from the interiors of grana thylakoid because of their large stroma-facing subunits (Dekker and Boekema, 2005). The localization of cytochrome *b_6_f *complex has proven to be controversial but is generally recognized to be between the stacked and unstacked regions (Nevo et al., 2012). It has also been postulated that access of cytochrome *b_6_f* to the grana core is regulated by the stromal gap (Kirchhoff et al., 2017).

PSII-LHCBII complexes and CURVATURE THYLAKOID1 (CURT1) appear to be important for stack formation in thylakoids (Armbruster et al., 2013). Cryo-ET revealed the arrangement of PSII and its associated proteins in thylakoid membranes of spinach and pea (Daum et al., 2010). The interactions between PSII and its LHCB subunits appear to mediate the stacking of thylakoid membranes. The small CURT1 proteins are embedded in the sharply curved membrane at the edge of grana stacks in *Arabidopsis*. The abundance of CURT1 proteins leads to narrow grana stacks with more layers, whereas the absence of CURT1 results in large increases of grana diameters and suppresses grana stacking (Pribil et al., 2018).

The protein complexes of the chloroplast electron transport chain require lipid membranes for chemiosmosis. Four lipids, monogalactosyldiacylglycerol (MGDG), digalactosyldiacylglycerol (DGDG), sulfoquinovosyldiacylglycerol (SQDG), and phosphatidylglycerol (PG), are the major lipid species of the thylakoid membrane. The cone-shaped MGDG is a non-bilayer forming lipid; DGDG, SQDG, and PG are bilayer forming lipids (Rocha et al., 2018). An *in vitro* study of thylakoid bilayers of pea chloroplasts showed that MGDG promotes membrane appression within the grana stacks and possibly stabilizes the inner membrane leaflet of the curve margins within each grana disc (Seiwert et al., 2018). DGDG is proposed to be involved in membrane stacking *via* hydrogen bonds between adjacent bilayers (Demé et al., 2014). It was shown that thylakoid lipid synthesis and photosynthetic gene regulation are coordinated by analysis of a mutant with severe loss of galactolipids (Kobayashi et al., 2013).

## Ultrastructural Analyses of Chloroplast Biogenesis

The hallmark of chloroplast biogenesis is construction of thylakoids in the stroma (Pogson et al., 2015). The proplastid is a small undifferentiated plastid with no internal structures except for residual membranes. Proplastids in the seed transform directly into chloroplasts if the seed germinates and grows under light, and the thylakoid assembly process during this transformation has been examined with ET (Liang et al., 2018). Soon after germination, the tubulovesicular residual membranes in the proplastids expand and then become flattened. This transformation requires binding of polysomes on their surface, suggesting that the process involves insertion of thylakoid membrane proteins. Subsequently, these monolayered membrane sheets merge into a single 3D network spanning the length of the plastid, and small stacks consisting of two or three layers derived from polysome-coated buds appear at many sites in the network. These pro-granal stacks gradually mature into full-fledged grana stacks. RNA-seq and immunoblot analysis revealed that PSII is the first of the major protein complexes to be established in the thylakoid membrane in proplastids of germinating *Arabidopsis* cotyledons (Liang et al., 2018).

Proplastids in the seed first develop into etioplasts if germinated under darkness. Etioplasts contain materials necessary for constructing thylakoids so that they can rapidly reshape into chloroplasts when light becomes available (Rudowska et al., 2012). Light-induced chloroplast biogenesis was examined in chemically fixed bean leaf samples with ET by Kowalewska et al. (2016) They showed that the paracrystalline prolamellar bodies lose lattice-like regularity upon illumination to become planar precursors of thylakoids and that grana stacks arise from the flattened membranes. Spectral and electrophoretic analyses indicated that PSII accumulation coincides with appearance of grana stacks in developing chloroplasts and that subunits of PSI and PSII are detected simultaneously (Kowalewska et al., 2016). 3D architectures of prolamellar bodies and intermediates of their transformation into thylakoids have not been determined in cryopreserved samples yet.

Chloroplasts in true leaves arise from proplastids in the shoot apical meristem (SAM). Proplastids in SAM have more elaborate membrane elements than proplastids in the seed. Some of the membrane elements exhibit basic stack architectures that grow and fuse to construct the mature thylakoid network as the SAM gives rise to mature leaves (Charuvi et al., 2012). Interestingly, proplastids in the precursors of epidermal cells lose thylakoids, undergoing changes opposite from proplastids in other cells in the SAM. It has not been examined whether epidermal cells in germinating cotyledon exhibit cell type-specific plastid transformations.

One of the characteristics of the proplastid-to-chloroplast conversion is that discrete thylakoid present in proplastids become interconnected to constitute the organelle-wide membrane network in the mature chloroplast. Individual thylakoids remain unconnected in chloroplasts of *fzl* mutant plants (Liang et al., 2018), indicating that FZL, which is an *Arabidopsis* dynamin targeted to the stroma (Gao et al., 2006), is involved in thylakoid membrane fusion. The animal and fungal Fzo dynamins mediate fusion of mitochondrial outer membranes in the cytosol (Breckenridge et al., 2009; Wang et al., 2016). Despite differences in substrates and sites of action, fusion-promoting functions of the dynamin family proteins appear to be conserved across the kingdoms.

## Dimorphic Chloroplasts in Single-Cell C4 Plants and Maize

C4 plants have two types of chloroplasts: one for fixing CO_2_ and the other that allows Rubisco to function at increased CO_2_ partial pressure (Edwards et al., 2001). The functional differences are linked to differential energy requirements and the distinction is illustrated in their thylakoid architectures visualized by TEM. One group of chloroplasts run linear electron flow consisting of both PSII and PSI to produce ATP and reducing power. Because PSII-LHCII complexes in the opposite thylakoid membranes can bind to each other, these chloroplasts have grana stacks and interconnecting stroma lamellae. The second type produce primarily ATP *via* cyclic electron flow through PSI and grana stacks are rare in these chloroplasts (Langdale et al., 1988; Evert et al., 1996). The maize is a C4 plant with Kranz anatomy in which functionally distinct chloroplasts are separately located in the mesophyll cell (MC) and in the bundle sheath cell (BSC) and they exhibit dimorphism related to the differential energy requirements (Sedelnikova et al., 2018).

In SCC4 plants, the two classes of chloroplasts are spatially segregated within a single cell (Freitag and Stichler, 2002; Akhani et al., 2005; Akhani et al., 2012). At least four species, *Suaeda aralocaspica, Bienertia cycloptera*, *Bienertia sinuspersici*, and *Bienertia kavirense*, have been found to exhibit single-cell C4 photosynthesis (Sharpe and Offermann, 2013). Intra-cellular organization of the dimorphic chloroplasts differs dramatically between the *Suaeda*-type and the *Bienertia*-type plants. In *Suaeda*-type SCC4 plants, the dimorphic chloroplasts are organized into distal and proximal chloroplasts at the poles of the cell, whereas in *Bienertia*-type SCC4 plants there are central and peripheral chloroplasts (Koteyeva et al., 2016).

**Figure 1:**
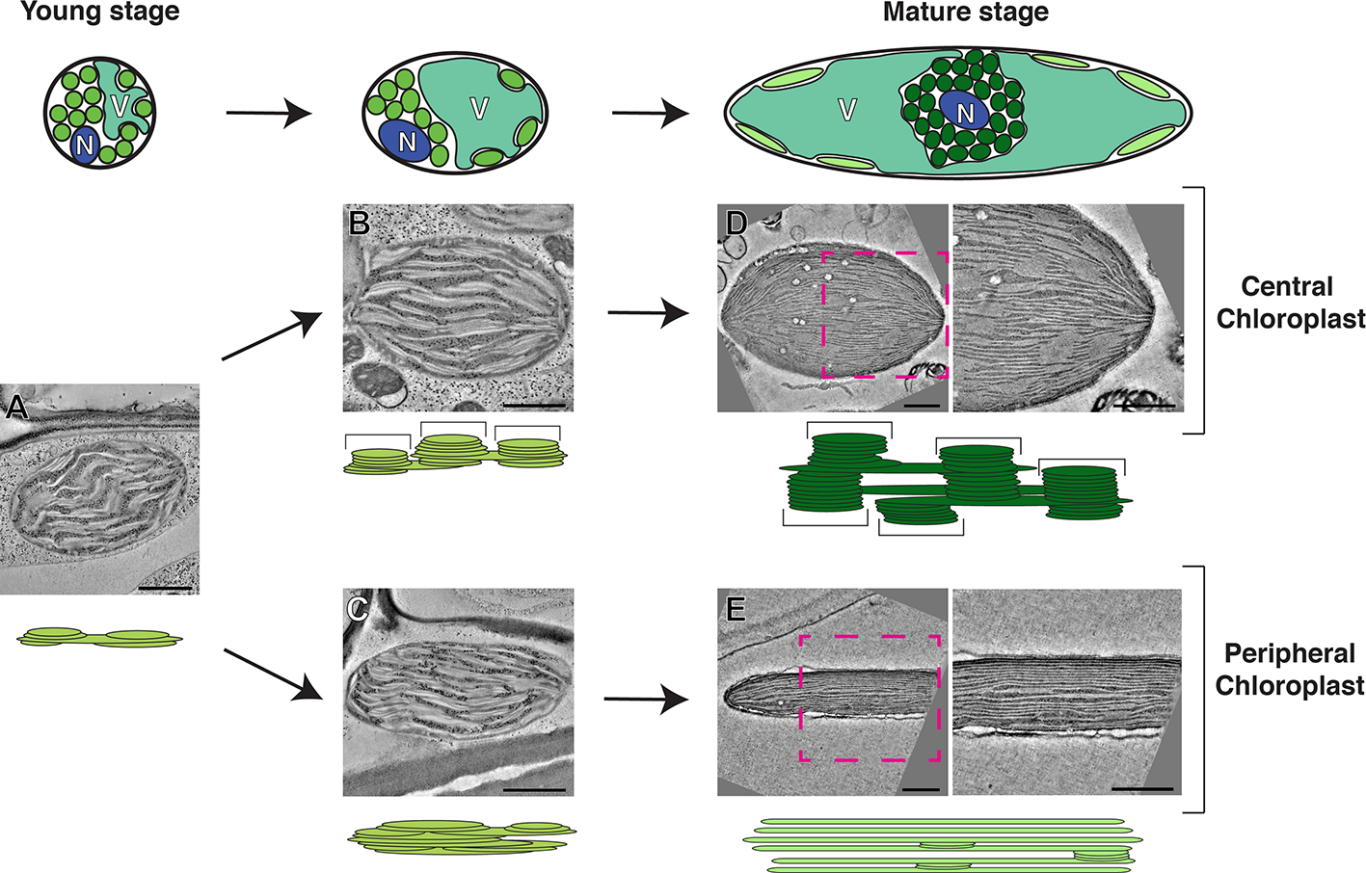
Model of the development of photosynthetic cells and their dimorphic chloroplasts in SCC4 plant *Bienertia sinuspersici*. Cells at the young stage (left) are small and round. Their cytoplasm has not been divided into central and peripheral regions because their vacuoles have not developed. Chloroplast in the young stage cells have simple thylakoid network with primitive grana stacks **(A)**. Chloroplasts are being segregated in the intermediate stage cells as the vacuole enlarges (middle). Grana stacks (brackets in the diagram) are discerned from unstacked lamellae in the central chloroplasts **(B)** while thylakoids in the peripheral chloroplast are more intertwined, making it difficult to distinguish grana stacks from stroma thylakoid **(C)**. In the mature stage (right), the cells have enlarged and most of the volume of the cell consists of a single vacuole. The central chloroplast (dark green) has taken on an ovoid shape with clearly delineated tall grana stacks **(D)**. The peripheral chloroplast (light green) has been flattened into discus shape. The majority of the thylakoid network consists of single-layer stroma lamellae with a smaller number of simple grana stacks **(E)**. For each stage, thylakoid models and electron tomographic slice images of corresponding chloroplasts are shown. N, nucleus; V, vacuole. Bars indicate 500 nm. The model is based on data published in Akhani et al. (2005); Koteyeva et al. (2016), and Mai et al. (2019).

*B. sinuspersici* has an NAD-ME type C4 cycle with peripheral and central chloroplasts that are functionally analogous to chloroplasts in BSC and MC, respectively, in Kranz-type C4 species (Offermann et al., 2011; Koteyeva et al., 2016; Ludwig, 2016; Rao and Dixon, 2016). The central chloroplasts in are arranged in an ovoid shape, and their thylakoids consist of grana stacks and unstacked stroma thylakoids (**Figure 1**). Their peripheral chloroplasts are disk-shaped and oppressed within the thin cytoplasmic gap between the vacuolar membrane and the plasma membrane. Stacked thylakoids are rarer in the peripheral chloroplast (**Figure 1**). Linear electron flow necessary for production of NADPH and ATP occurs in the thylakoids of central chloroplasts, whereas peripheral chloroplasts use ATP to convert CO_2_ into aspartates. By contrast, the C4 biochemistry in maize is primarily of the NADP-malic enzyme (NADP-ME) type in which chloroplasts in MCs need both PSII and PSI for linear electron flow (Arrivault et al., 2017).

## Biogenesis of Dimorphic Chloroplasts in a Single-Cell C4 Plant, *Bienertia sinuspersici*

Among SCC4 plant species, photosynthesis of *B. sinuspersici* has been studied most thoroughly (Minges et al., 2019). The development of *B. sinuspersici* photosynthetic apparatus happens along a gradient with the younger chlorenchyma cells at the leaf base and the more mature cells at the tip (Lara et al., 2008). In the young cells, C3 photosynthesis occurs, and all chloroplasts have similar levels of *Rubisco* mRNA. Selective enrichment of Rubisco in the central chloroplast cluster is a key C4 characteristic, and this feature emerges in a longitudinal progression of the chlorenchyma cell maturation (Koteyeva et al., 2016). Park et al. have classified the *B. sinuspersici* SCC4 cell maturation into four stages (Park et al., 2008). In the youngest stage, no distinction between central and peripheral chloroplasts is observed. In later stages, the chloroplasts are separated by growing vacuoles until there is a clear central chloroplast compartment containing tightly packed central chloroplasts, almost all mitochondria, and the nucleus. Transcripts encoding *Rubisco* are concentrated in the central domain in the mature chlorenchyma cells (Voznesenskaya et al., 2005; Koteyeva et al., 2016). The positioning of the peripheral chloroplasts is dependent upon the cytoskeleton, which maintains the spatial integrity of the SCC4 structure (Chuong et al., 2006).

The biogenesis of dimorphic chloroplasts in SCC4 plants requires differential targeting of nucleus-encoded chloroplast proteins to the two types of chloroplasts. Theories for explaining how proteins from nuclear encoded genes are selectively targeted include selective protein degradation, mRNA targeting, and selective import (Offermann et al., 2015; Erlinghaeuser et al., 2016). Wimmer et al. provided evidence that transit peptides of *B. sinuspersici* chloroplast proteins have two elements, one for general chloroplast import and the other for selective targeting (Wimmer et al., 2017). Plastid-targeted pyruvate orthophosphate-dikinases (PPDKs) in C4 plants regenerate phosphoenolpyruvate, the CO2 acceptor in C4 cycle. By contrast to PPDKs in C4 plants, functions of plastid-targeted PPDKs in C3 plants have not been characterized well (Chastain et al., 2011). *B. sinuspersici *chlorenchyma cells have PPDKs in* *their peripheral chloroplasts; the specific localization requires a sequence element in PPDK that blocks its entry into central chloroplasts. A recent electron tomography (ET) imaging of *B. sinuspersici* visualized the process of chloroplast differentiation (Mai et al., 2019). Young chloroplasts have grana stacks and stroma lamellae and they are not morphologically distinguishable before spatially separated by the central vacuole. Grana stacks of chloroplasts located in the cell center multiply and each acquires more layers. By contrast, grana stacks displaced to the cell periphery stretch out, gradually losing the stack architecture to the degree that most thylakoids become monolayered. The process of dimorphic chloroplast biogenesis in *B. sinuspersici* is summarized in **Figure 1**. It was suggested that invaginations from the inner envelope membrane supply materials for thylakoid expansion because the membrane ingrowths stretch out to become associated with existing thylakoids.

In addition, TEM/ET study of *B. sinuspersici* chloroplasts revealed how thylakoids are partitioned in dividing chloroplasts. It is difficult to capture dividing chloroplasts by TEM because chloroplasts divide less frequently than proplastids in the meristematic cells. Dozens of chloroplasts are clustered in *B. sinuspersici* cells and they proliferate by binary fission. In ET slice images of chloroplast undergoing fission, thylakoids at the division plane were severed before the envelopes were squeezed (Mai et al., 2019). Interestingly, PSII complexes are dislocated from the division site probably because they constitute closely packed 2D array that could block thylakoid severing. It has been shown that two ring complexes, one ring on outer envelope membrane and the other ring inside the inner envelope membrane assemble at the midzone of a dividing plastids to constrict the envelope membranes (Osteryoung and Pyke, 2014). GTPases, including DRP5B and FtsZ, and regulatory proteins were shown to constitute the ring complexes. However, no information about the molecular machinery constricting thylakoids is available (Chen et al., 2018).

## Conclusions and Future Prospects

Use of ET and cryo-preservation techniques has led to a better understanding of chloroplast structures and their biogenesis. Correlative gene expression analyses, biochemical analyses, and photosynthetic activity measurements provided further insight into the molecular mechanisms of thylakoid assembly. Novel membrane elements in the chloroplast will be uncovered in cryo-fixed plant cell samples (Kirchhoff, 2018). Intermediates of membrane assembly that were not previously documented are discerned in high-pressure frozen plant cell samples because these structures are not lost during chemical fixation that takes minutes to hours (Staehelin and Kang, 2008; Wang et al., 2017; Karahara and Kang, 2013; Wang et al., 2019). For example, the kinked thylakoid membranes at the midplane of dividing chloroplasts have not been reported until Mai et al. (2019) where photosynthetic cells of *Arabidopsis* and *B. sinuspersici* were preserved by high pressure freezing. Combined with ET, transient structures in the thylakoid during its partitioning was revealed at nanometer-level resolutions.

**Figure 2:**
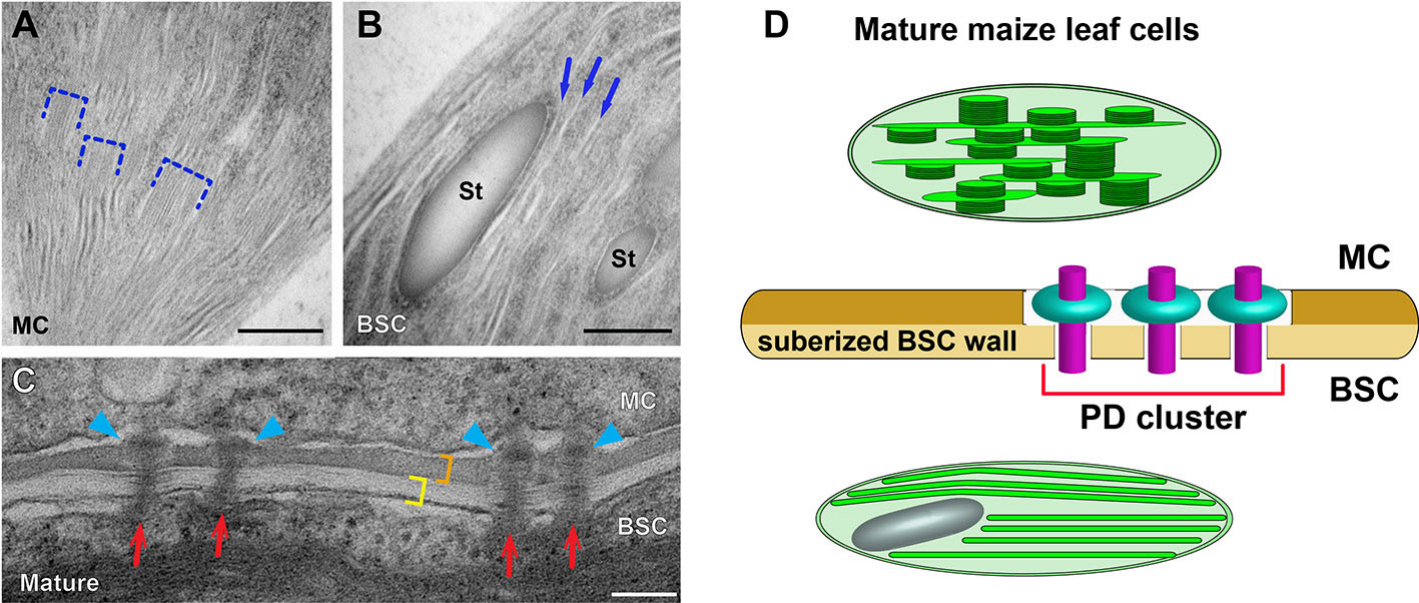
Dimorphic chloroplasts and plasmodesmata in Kranz anatomy of the maize leaf. **(A, B)** Transmission electron micrographs of a mesophyll cell (MC) chloroplast and a bundle sheath cell (BSC) chloroplast. Grana stacks (dashed brackets) are abundant in the MC chloroplast **(A)**. Thylakoids are monolayered in the BSC chloroplast **(B)**. Three unstacked stroma lamellae are marked with arrows in B. **(C)** Plasmodesmata (PD) at the MC-BSC interface of mature **(D)** maize leaf tissues. PD (red arrows) in the mature tissue have “sphincter” rings in the MC side wall (blue arrowheads). The BSC wall (yellow bracket) is suberized and it is stained differentially from the MC wall (orange bracket). **(D)** A diagram illustrating the dimorphic chloroplasts and PD in the maize leaf based on data published in 1996; Evert et al. (1977); Danila et al. (2016), and Mertz and Brutnell (2014). MCs and BSCs are linked *via* clusters of specialized PD that transverse a suberized cell wall. St: starch particle. Scale bars in **(A, B)** and **(C, D)** indicate 500 nm, and 100 nm, respectively.

Given the significance of maize as a crop plant, it is reasonable that the development of C4 cycle in BSC and MC has been characterized along the developmental gradient of the maize leaf blade by means of proteomic and transcriptomic approaches (Li et al., 2010; Majeran et al., 2010; Tausta et al., 2014; Wang et al., 2014). Majeran et al. (2010) carried out comparison of chloroplast structures in BSCs and MCs with conventional TEM after chemical fixation for correlating the morphological diversification with cell type-specific accumulation of thylakoid membrane proteins. ET and high-pressure freezing have not been adopted for investigating structural dynamics involved in the biogenesis of maize dimorphic chloroplasts at multiple developmental stages of Kranz anatomy. It remains elusive how grana stacks assemble and proliferate in MC chloroplasts while they degenerate in BSC chloroplasts. Dual cell C4 cycle employs symplastic transport and it has been demonstrated that plasmodesmata (PD) connecting MC and BSC in the mature maize leaf cluster to form pitfields (Danila et al., 2016) and they are equipped with a unique “sphincter” module (Evert et al., 1977; Bilska and Sowinski, 2010). It is not understood whether the attachment of PD is involved in the maize C4 cycle and regulation of C4 photosynthesis (**Figure 2**). We expect that advanced electron microscopy methods will continue to reveal novel aspects of the structural biology of the C4 photosynthetic machinery.

## Author Contributions 

KM and PG prepared figures. KM, PG, and B-HK wrote the manuscript. All authors contributed to the article and approved the submitted version.

## Funding 

We are grateful for support from Rural Development Administration of Korea (Project No. 10953092019), Hong Kong Research Grant Council (GRF14121019, GRF14126116, AoE/M-05/12, C4002-17G), and Chinese University of Hong Kong (Direct Grants).

## Conflict of Interest

The authors declare that the research was conducted in the absence of any commercial or financial relationships that could be construed as a potential conflict of interest.
